# Relation Between Unconjugated Bilirubin and Peripheral Biomarkers of Inflammation Derived From Complete Blood Counts in Patients With Acute Stage of Schizophrenia

**DOI:** 10.3389/fpsyt.2022.843985

**Published:** 2022-04-07

**Authors:** Haiting Xu, Yanyan Wei, Lina Zheng, Hua Zhang, Tangren Luo, Hongjuan Li, Jinbao Ma, Jingxu Chen

**Affiliations:** ^1^Peking University HuiLongGuan Clinical Medical School, Beijing HuiLongGuan Hospital, Beijing, China; ^2^Liaocheng People's Hospital, Liaocheng, China; ^3^Dongying People's Hospital, Dongying, China; ^4^The Third Hospital of Longyan, Longyan, China; ^5^Capital Medical University Beijing TongRen Hospital, Beijing, China

**Keywords:** schizophrenia, oxidative stress, inflammation, unconjugated bilirubin, peripheral biomarkers of inflammation

## Abstract

**Background:**

Inflammation and oxidative stress are the major leading hypothetical causes of schizophrenia. Unconjugated bilirubin (UCB) is an efficient endogenous plasma antioxidant. Inflammation is closely linked to oxidative stress. The relationship between UCB and inflammatory markers should be paid close attention in schizophrenia acute stage. In this paper, combined UCB and inflammatory markers were evaluated for their capability in predicting schizophrenia in the acute stage to find an easy and effective indicator to identify acute schizophrenia.

**Methods:**

A total of 6,937 acute schizophrenia patients and 6,404 healthy controls (HCs) were enrolled. UCB and peripheral biomarkers of inflammation derived from complete blood counts (CBC) were investigated in the subjects with acute schizophrenia, and the results were compared with HCs. Simultaneously, Spearman test was employed to assess the correlation between the variables, while logistic regression was adopted to determine the combined equation and receiver operating characteristic curve was used to evaluate the combined value of UCB and peripheral biomarkers of inflammation derived from CBC to predict schizophrenia in the acute stage.

**Results:**

The study indicates that white blood cells, neutrophil, monocyte, mean platelet volume (MPV), red cell distribution width (RDW), neutrophil/lymphocyte ratio (NLR), and monocyte/lymphocyte ratio (MLR) have significantly increased in schizophrenia (*p* < 0.05 for all), while platelet, lymphocyte, and platelet/lymphocyte ratio (PLR) in schizophrenia have significantly decreased (*p* < 0.05 for all). UCB exhibits negative correlation with MPV significantly (*r* = 0.121, *p* < 0.001), and no correlation with neutrophil and monocyte. The correlations between UCB and other peripheral biomarkers of inflammation derived from CBC are very weak. MPV, RDW, NLR, MLR, PLR, and UCB were taken as independent variables for a logistic regression analysis. The model was as follows:

$$\begin{array}{l} \text{Logit }\left(\text{P}1\right)=-6.141+0.827\text{ MPV}+5.613\text{ MLR}-0.005\text{ PLR}-0.346\text{ UBC}. \end{array}$$

The combination demonstrates better effectiveness in predicting schizophrenia in the acute stage (AUC 0.831, 95% CI 0.825 to 0.837).

**Conclusion:**

UCB has a protective effect on acute stage of schizophrenia, which is weak and indirect by affecting the proinflammatory processes. Our findings suggest that a combination of MLR, MPV, PLR, and UBC could be used to predict acute stage of schizophrenia.

## Introduction

Schizophrenia is a serious chronic mental disorder, which can cause various symptoms such as delusions, hallucinations, and cognitive impairments (1). It is a debilitating psychosis with ~1% global lifetime risk (2). The disease often causes patients to have difficulties in various aspects of their daily lives including social, occupational, and general functioning (3, 4). Furthermore, although the etiology of schizophrenia is unknown, the genetic liability of disabling conditions has been reported (5), which has affected perception of patients with schizophrenia, exaggerated the dangerousness and unpredictability, and increased patients' stigma (6). Scientists have been trying for a long time to investigate the pathogenesis of schizophrenia and search for biomarkers that may enable the identification of disease with objective diagnostic criteria (7).

Unconjugated bilirubin (UCB) is the water-insoluble fraction of total bilirubin in the serum, which has been found to have powerful anti-inflammatory capacities (8) and plays a role in antioxidant in oxidative stress–related diseases in low concentrations (i.e., 20–100 μM) (between 1.17 and 5.85 mg/dl) (9), while moderate and high levels directly cause cell apoptosis (10). An animal study suggested that UCB might be important to the pathophysiology of schizophrenia (11). Many clinical studies have observed a correlation between bilirubin and schizophrenia, however, with discrepancies in the results. A research conducted by Gama-Marques et al. (12) found higher levels of UCB associated with an increased incidence of acute schizophrenia. Lu et al. (13) reported in their examination that peripheral TBIL of schizophrenic patients in the acute and remission stages were higher than those of healthy controls. On the contrary, Yin et al. (14) found that serum bilirubin concentration was significantly decreased in schizophrenia patients compared with healthy controls. A recent study conducted by Becklen et al. (15) set out to investigate the levels of total plasma bilirubin in subjects with a first-episode psychosis, and found that bilirubin levels were reduced compared with 20 healthy controls. The results of these existing studies are inconsistent, and there have been relatively few studies with large sample sizes about the relationship between bilirubin and schizophrenia.

Many studies have focused on the relationship between inflammation and the pathogenesis of schizophrenia (16). The platelet/lymphocyte ratio (PLR), neutrophil/lymphocyte ratio (NLR), monocyte/lymphocyte ratio (MLR), and red cell distribution width (RDW) are reproducible biomarkers of inflammation and calculated from the complete blood count, and its pathogenetic role has been investigated in schizophrenia (17). Elevated levels of these markers have been reported in schizophrenia (18, 19).

Inflammation is a complex pathophysiological process relying on multiple biological pathways and has high correlation with oxidative stress (20). In recent years, peripheral biomarkers of inflammation derived from CBC have been reported to reflect the condition of central oxidative stress in schizophrenia (21). Correlation between bilirubin and inflammatory markers is reported to exist, in that UCB appears to be consumed by the increased inflammation levels (22). There could be an important underlying link in the association between UCB and inflammatory markers. In this study, we hypothesize that there might be a negative association between UCB and inflammatory markers. So far, the relationship of UCB and inflammatory markers has not been looked into in psychiatric disorders. We aim to investigate the changes of UCB and peripheral biomarkers of inflammation derived from CBC in acute schizophrenia with large samples, and to confirm the relationship between them. This study may provide some clues to understanding the mechanism of schizophrenia, which may facilitate finding new ways of protecting schizophrenia.

Complete blood counts and UCB are biomarkers that can be obtained quickly in routine blood tests. We hypothesize that inflammatory process and oxidative stress injury might be combined to get more information for predicting schizophrenia in the acute stage. Therefore, in this study we combine peripheral biomarkers of inflammation derived from CBC (inflammatory biomarkers) and UCB (an oxidative stress biomarker) into parameters using a simplified equation to assess the capability of combined information to predict schizophrenia in the acute stage. In this way, we hope to find a simple and reliable auxiliary index to identify acute schizophrenia, hence facilitate diagnosis of the disease, which is typically difficult due to the lack of objective diagnostic criteria for schizophrenia.

## Methods

### Subjects and Data Collection

This study was approved by the Local Ethics Committee of Hui-Long-Guan Hospital. The study was designed as a cross-sectional, retrospective study. The research was conducted in Beijing Hui-Long-Guan hospital, in collaboration with Clinical Medical College of Peking University, China in a six-year period from March 15, 2015 to March 15, 2021.

The research data collection was based on the HIS System (a database used in Hui-Long-Guan Hospital). The data were extracted from the electronic medical records of the hospital without any accessible personal identifying information of the patients (except their hospital registration numbers). Our inclusion criteria looked for all with acute psychosis under the diagnosis of schizophrenia disorder diagnosed by two independent senior psychiatrists according to the International Classification of Diseases-10 (ICD-10 codes between F20.0 and F20.9). Aged between 18 and 65 years, admitted to our psychiatric ward, all subjects were Han Chinese. Exclusion criteria include presently comorbid with any other psychiatric disorder, presence of a physical disease that may alter inflammatory status or serum bilirubin concentrations such as hepatic or renal failure, diabetes mellitus, hypertension, acute infection, acute or chronic immuno-inflammatory disease or pregnancy, obesity or being underweight (body mass index >29.9 or <18.5 kg/m^2^, respectively), heavy smoking (>20 cigarettes per day), being under an anti-inflammatory or immunosuppressive medication, documented laboratory findings of liver or renal pathology, iron-deficiency anemia, and lack of laboratory screening at admission. The control group was recruited from the local community visited outpatient unit for healthy medical examinations, coded with ICD-10 Z00.00 (encounter for general adult medical examination without abnormal findings), without any record of personal or family history of psychiatric disorders. The control group were Han Chinese. In total, 6,404 healthy controls were included at age of 18–65 years. All subjects signed consent, which was approved by the Institutional Research Ethics Committee of Beijing Hui-Long-Guan Hospital.

**Figure 1:**
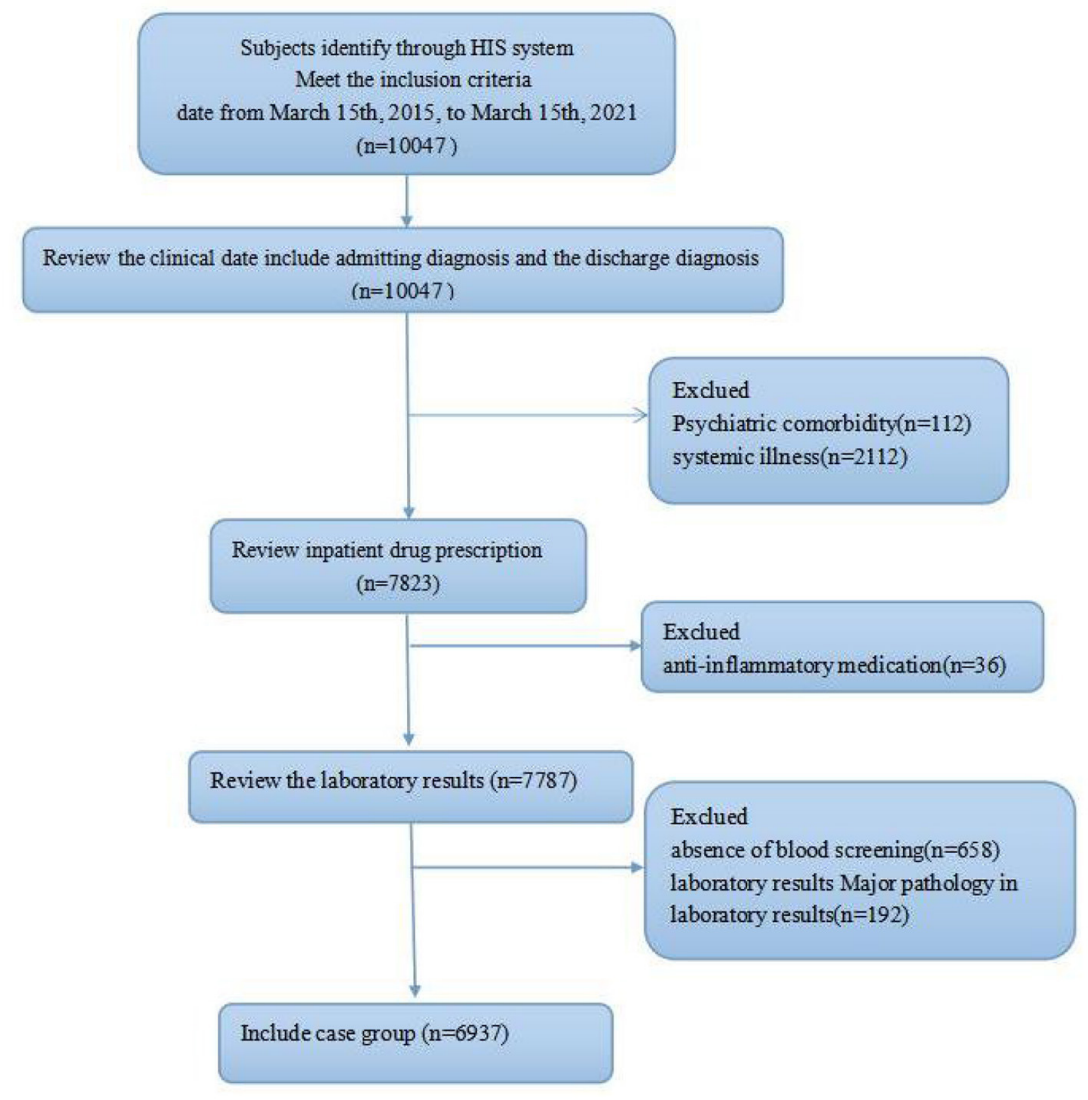
Flow diagram of the patient selection process, showing the study inclusion and exclusion criteria.

After the eligibility evaluation, the patients' electronic and hard-copy files were reviewed, and patients' hospital registration numbers were provided. According to the hospital protocol, blood sampling was performed after an overnight fast. Preliminarily, data of 10,047 patients who were screened within the designated time frame were reviewed. A total of 112 patients were excluded due to present comorbidity with other psychiatric disorders, 2,112 excluded for presence of a physical comorbidity, 36 excluded due to being under an anti-inflammatory medication, 658 excluded due to absence of blood screening, and 192 excluded for major pathology in laboratory results. Also, 6,937 subjects that age, complete blood count, and unconjugated bilirubin recorded were included, as shown in the flow process in Figure 1.

### Statistical Analysis

SPSS (Statistical Package for the Social Sciences ver. 25.0; SPSS Inc., Chicago, Illinois, USA) computer program was used for statistical analysis. The variables were analyzed using an analytical method, i.e., Kolmogorov–Smirnov test, to determine whether they follow normal distribution. Spearman correlation was selected because the data did not conform to normal distribution. For comparison between the groups, Mann–Whitney *U*-test was used. A logistic regression model (forward stepwise selection) was adopted to estimate the potential predictive value of PLR, MLR, PLR, RDW, MPV, and UCB in patients with schizophrenia. Quantitative variables were expressed in the manner of mean ± SD. All statistical tests were 2-tailed and *p*-value < 0.05 was considered as statistically significant. Logistic regression was employed to determine the combined equation. Receiver operating characteristic (ROC) curve was adopted to assess the combined value of UCB and peripheral biomarkers of inflammation derived from CBC to predict schizophrenia in the acute stage.

## Results

The characteristics of the study population are summarized in Table 1. There were 6,937 subjects with schizophrenia and 6,404 healthy controls. The ages of the schizophrenia and control groups were 39.410 ± 12.461 and 39.380 ± 9.751 years, *p* > 0.05 (Table 1). Notably, patients with schizophrenia had significantly lower UCB compared with healthy controls (*p* < 0.05 for all). Besides, consistent with previous studies (19, 23), our study shows that WBC, neutrophil, monocyte, MPV, RDW, NLR, and MLR have significantly increased in patients with schizophrenia (*p* < 0.05 for all), while platelet, lymphocyte, and PLR in patients with schizophrenia have significantly decreased (*p* < 0.05 for all).

**Table 1:** Baseline characteristics of the study population.

	**Schizophrenia (***n*** = 6,937)**	**Control (***n*** = 6,404)**	**χ^2^/** ***Z***	***P*** **-value**
Age (years)	39.41 ± 12.46	39.38 ± 9.75	1.47	0.14
Male, n (%)	2,773 (39.97)	2,483 (38.24)	2.03	0.16
UCB (μmol/L)	8.31 ± 2.34	10.62 ± 2.75	44.53	<0.001
WBC (k/μl)	6.25 ± 1.40	6.14 ± 1.51	5.08	<0.001
Plt (k/μl)	238.59 ± 51.62	257.39 ± 57.71	17.16	<0.001
Lymphocyte (k/μl)	1.98 ± 0.58	1.99 ± 0.52	2.34	0.02
Neutrophil (k/μl)	3.69 ± 1.18	3.58 ± 1.17	5.15	<0.001
Monocyte (k/μl)	0.43 ± 0.14	0.40 ± 0.12	11.68	<0.001
MPV	9.74 ± 1.08	8.69 ± 1.11	52.40	<0.001
RDW	13.52 ± 1.63	13.28 ± 1.19	7.42	<0.001
NLR (%)	2.05 ± 1.04	1.89 ± 0.73	5.22	<0.001
MLR (%)	0.23 ± 0.1	0.21 ± 0.07	12.52	<0.001
PLR (%)	130.26 ± 47.43	136.71 ± 43.81	10.49	<0.001

*UCB, unconjugated bilirubin; Plt, platelets; WBC, white blood cell; MPV, mean platelet volume; RDW, red cell distribution width; NLR, neutrophil/lymphocyte ratio; MLR, monocyte/lymphocyte ratio; PLR, platelet/lymphocyte ratio*.

*Mann–Whitney U-test for abnormally distributed data. Values are given as mean ± SD*.

To explore the relationship between serum bilirubin concentration and peripheral biomarkers of inflammation derived from CBC, Spearman correlation analyses were performed. As shown in Table 2, UCB (*r* = 0.121, *p* < 0.001) were found to be negatively correlated with MPV significantly. However, there were no correlations between UCB concentrations and neutrophil or monocyte. The correlations between UCB and other peripheral biomarkers of inflammation derived from CBC are very weak, i.e., UCB is negatively correlated with lymphocyte (*r* = −0.033, *p* < 0.001), platelet counts (*r* = −0.05, *p* < 0.001), RDW (*r* = −0.041, *p* < 0.001), and WBC (*r* = −0.023, *p* = 0.05), and positively correlated with NLR (*r* = 0.03, *p* = 0.011) in the subjects with schizophrenia.

**Table 2:** Correlations between bilirubin and peripheral biomarkers of inflammation derived from CBC in schizophrenia.

	**UCB**	
	***r***	***p***
Age	−0.008	0.280
Neutrophil	0.002	0.885
Lymphocyte	−0.033**	0.006
Monocyte	−0.005	0.678
Plt	−0.056**	0.00
RDW	−0.045**	0.00
MPV	−0.122**	0.00
NLR	0.030*	0.011
MLR	0.022	0.059
PLR	−0.007	0.522
WBC	−0.021	0.081

*UCB, unconjugated bilirubin; Plt, platelets; WBC, white blood cell; MPV, mean platelet volume; RDW, red cell distribution width; NLR, neutrophil/lymphocyte ratio; MLR, monocyte/lymphocyte ratio; PLR, platelet/lymphocyte ratio*.

* *P < 0.05*;

** *P < 0.01*.

Given that the MPV, RDW, MLR, NLR, PLR, and UCB were different between the control group and the acute schizophrenia group, we sought to determine whether these inflammation-based markers and UCB could be predictors of schizophrenia. We used schizophrenia and control as dependent variables for the stepwise logistic regression model. The model was run to assess the potential predictive values of serum bilirubin and peripheral biomarkers of inflammation derived from CBC. The combined equation of the logistic regression model was *logit*(*P*1) = −6.141+0.827 *MPV*+5.613 *MLR*−0.005 *PLR*−0.346 *UBC* (Table 3); therefore, the “combined” value refers to *logit*(*P*1) in our report.

**Table 3:** The combined equation of logistic regression model with risk factors of schizophrenia compared with healthy control groups.

	**β**	**SE**	**Wald**	**df**	***p***	**OR**	**OR 95% Cl**
MPV	0.827	0.021	1,536.267	1	<0.001	2.286	2.194–2.383
PLR	−0.005	0.001	76.265	1	<0.001	0.995	0.994–0.996
MLR	5.613	0.310	327.261	1	<0.001	274.077	149.192–503.499
UCB	−0.346	0.009	1,444.671	1	<0.001	0.708	0.695–0.720
Constant term	−6.141	0.319	369.470	1	<0.001	0	–

*MPV, mean platelet volume; MLR, monocyte/lymphocyte ratio; PLR, platelet/lymphocyte ratio; UCB, unconjugated bilirubin*.

*Cox&Snell R^2^ = 0.313, Nagelkerke R^2^ = 0.418*.

MPV, RDW, MLR, NLR, and PLR were combined using logistic regression. The peripheral biomarkers of inflammation of logistic regression model were *logit* (*P*2) = −10.911+0.882*MPV*+0.166*RDW*+4.877*MLR*−0.003*PLR* (Table 4).

**Table 4:** Peripheral biomarkers of inflammation derived from CBC of logistic regression model with risk factors of schizophrenia compared with healthy control groups.

	**β**	**SE**	**Wald**	**df**	***p***	**OR**	**OR 95% Cl**
MPV	0.882	0.020	1,968.693	1	<0.001	2.416	2.323–2.512
PLR	−0.003	0.001	34.914	1	<0.001	0.997	0.996–0.998
RDW	0.166	0.015	121.358	1	<0.001	1.181	1.146–1.216
MLR	4.877	0.283	296.983	1	<0.001	131.231	75.361–228.521
Constant term	−10.911	0.293	1,389.401	1	<0.001	0	–

*MPV, mean platelet volume; RDW, red cell distribution width; MLR, monocyte/lymphocyte ratio; PLR, platelet/lymphocyte ratio*.

*Cox&Snell R^2^ = 0.214, Nagelkerke R^2^ = 0.286*.

**Figure 2:**
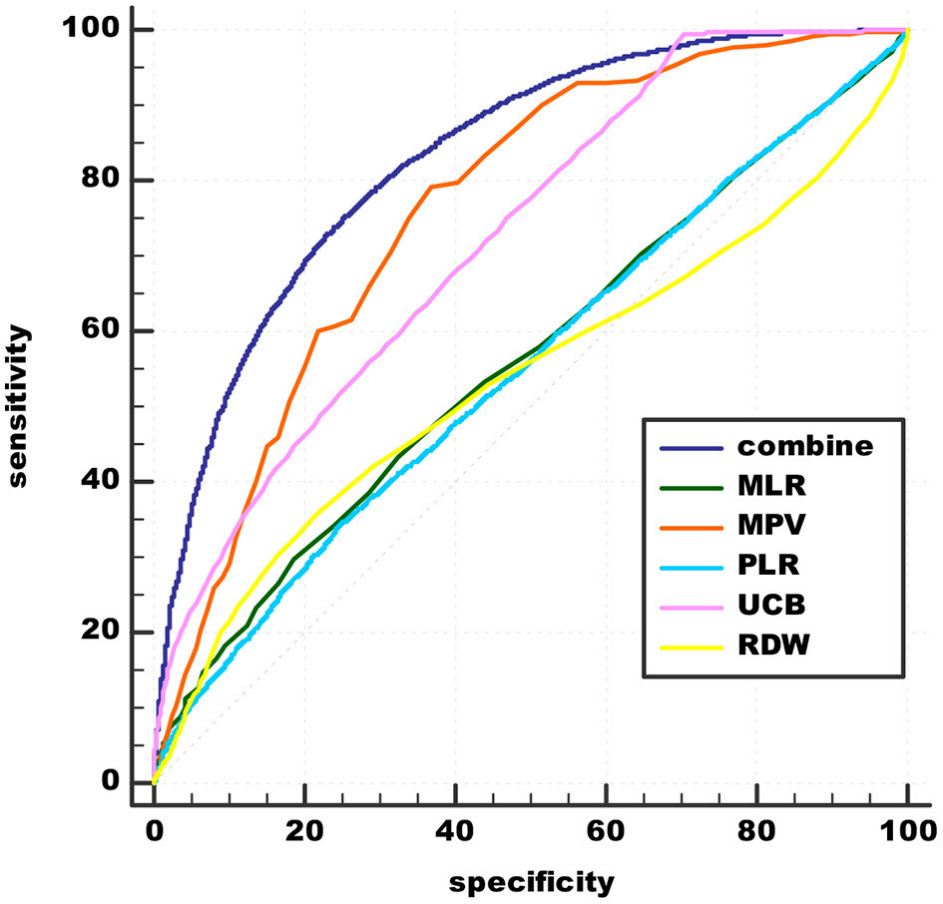
Admission of MLR, MPV, PLR, RDW, UCB, and combinations in predicting acute schizophrenia. UCB, unconjugated bilirubin; MPV, mean platelet volume; RDW, red cell distribution width; NLR, neutrophil/lymphocyte ratio; MLR, monocyte/lymphocyte ratio; PLR, platelet/lymphocyte ratio.

Using schizophrenia as a classification variable, ROC curves for the effectiveness of MPV, RDW, MLR, PLR, UCB, and the combined values of these variables in predicting acute schizophrenia are shown in Figure 2. The area under the ROC is shown in Table 5.

**Table 5:** Admission of peripheral biomarkers of inflammation derived from CBC and UCB as predictors for diagnosis of acute schizophrenia.

**Variables**	**Cut-off point**	**Sensitivity (%)**	**Specificity (%)**	**AUC (0–1.0)**	**95% CI**
MPV	8.8 fl	79.33	81.2	0.762	0.755–0.769
PLR	106.45	34.31	75.36	0.552	0.544–0.561
RDW	13.6%	35.84	78.31	0.537	0.529–0.546
MLR	0.25	29.97	81.20	0.562	0.554–0.571
UCB	12.0 mmol/L	99.63	29.53	0.723	0.715–0.730

**Figure 3:**
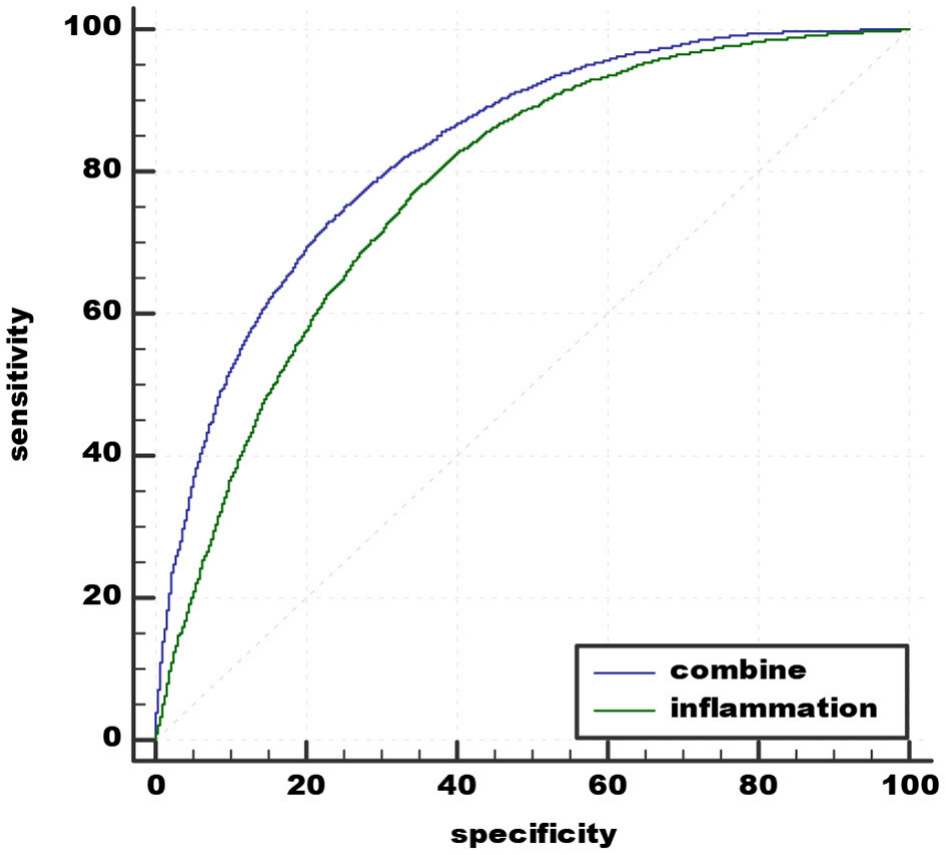
Admission of the combination of MLR, MPV, PLR, RDW, and the combination of MLR, MPV, PLR, RDW, and UCB in predicting acute schizophrenia. UCB, unconjugated bilirubin; MPV, mean platelet volume; RDW, red cell distribution width; NLR, neutrophil/lymphocyte ratio; MLR, monocyte/lymphocyte ratio; PLR, platelet/lymphocyte ratio.

A comparison was made between the new predictive model and inflammation predictive model. The best cut-off value of peripheral biomarkers of inflammation was 0.45959 with sensitivity 77.38% and specificity 65.57% (AUC 0.779, 95% CI 0.772–0.786). The recommended cut-off value for the combined value based on the maximum of Youden's index on the ROC curve was 0.53126 and it exhibits 73.01% sensitivity and 76.87% specificity in the prediction (AUC 0.831, 95% CI 0.825–0.837). The results prove that the combination of peripheral biomarkers of inflammation and UCB is more effective in predicting acute schizophrenia (Figure 3).

## Discussion

In this study, we found that there was a reduction in UCB in acute schizophrenia. Data from logistic regression further support the conclusion that UCB has a protection effect on schizophrenia in the acute stage of schizophrenia. However, we were the first to report a negative correlation between UCB and lymphocyte, platelet, MPV, and RDW, and a positive correlation between UCB and NLR in the acute stage of schizophrenia. We found that a combination of markers including MLR, MPV, PLR, and UBC could be leveraged as predictors for the diagnosis of acute schizophrenia.

There has been good amount of research on investigating the relationship between UCB and the incidence of schizophrenia that is extensive. However, it is not clear whether UCB concentration is positively or negatively associated with the incidence of schizophrenia. Gama-Marques et al.'s study (12) claimed that schizophrenia was linked to higher levels of UCB. However, Vitek et al. (24) claimed the opposite, i.e., schizophrenia was linked with lower levels of the molecule. Our study supports the latter. In our study, UCB levels are clearly lower in patients with acute psychotic episodes of the schizophrenia when compared with healthy controls. Pommerening Dornelles et al. (25) pointed out that studies reporting the lower UCB levels in schizophrenia (21, 24) could indicate a chronic proinflammatory state caused by elevated reactive oxygen species and the consumption of the antioxidant potential, hence leading to neuronal damage. The study presented in this paper is the first to investigate the relation between peripheral biomarkers of inflammation derived from CBC and UCB levels. We found that lymphocyte has a negative relationship with UCB. Keshavan et al. (26) reported that UCB has apoptotic pathways, primarily by binding to the mitochondrial membrane, resulting in the release of cytochrome *c* into the cytoplasm and activation of caspase-9. This may be the mechanism of the apoptotic effect of UCB in reducing lymphocyte counts (27). Naveen Kumar et al. (28) indicated that UCB has been shown to cause apoptosis of platelets *in vitro* at the concentration range 0–200 μM (0–11.7 mg/dl). One study (29) showed that reduced P-selectin expression, which is a protein released from activated platelets, was significantly linked to increasing bilirubin concentrations. MPV is an indicator of platelet activation. In our results, MPV and platelet counts are negatively correlated with UCB; hence, we speculate that there could be a vicious cycle between platelet activity and UCB in schizophrenia. It deserves further study that involves intermediates such as P-selection on the relationship between platelet counts, MPV, and UCB. RDW is considered as a comprehensive biomarker of chronic inflammation and oxidative stress (30). Elevated RDW reflects enhanced cellular oxidative stress, which could lead to the consumption and even depletion of natural antioxidants, thus resulting in decrease in UCB concentration. In this study, UCB was significantly negatively correlated with RDW levels in the schizophrenia group. However, the present study is a cross-sectional study; further prospective cohort studies are needed to explore the relationship between UCB and RDW in acute schizophrenia. We thought that the lymphocyte decrease caused by UCB slightly increases the level of NLR, leading to the positive correlation between UCB and NLR found in our study.

Inflammatory processes play a key role in the development of schizophrenia. The principal finding of the present study is significantly increased blood total WBC counts, neutrophils, and monocytes in patients with acute schizophrenia vs. controls. The finding is consistent with a previous meta-analysis research (23). In this study, we observed lower lymphocyte counts in the schizophrenia group compared with the control group. Semiz et al. (31) and Ozdin and Boke (32) found low lymphocyte counts in schizophrenia, which were also consistent with our study. Platelets are involved in serotonin (5-HT) hypothesis and inflammation hypothesis to explain the pathogenesis of schizophrenia (33). One study conducted in first-episode schizophrenia found that platelet count was significantly higher than that in the HCs (34). In Peitl et al.'s study (35), there was no statistically significant difference between schizophrenia and HC groups regarding total platelet count. Our results did not replicate the results, in which platelet counts were significantly lower in patients with schizophrenia than in healthy controls. There could be two reasons to explain the discrepancy. First, platelet is a simple cell without nuclei, which is sensitive and fragile, and tends to apoptose when undergoing stimuli (36) such as schizophrenia. The apoptosis of platelet transpires *via* augmented levels of reactive oxygen species (28, 37), which was supported by our study, where we found a negative relationship between platelet count and serum bilirubin that acts as a potent antioxidant. This may be the main mechanism of platelet count decline in our findings. Second, because platelets are very vulnerable to therapeutic drugs in the circulation (36), in our sample, patients were not taken off their antipsychotics, which is a confounding variable. Further study should rule them out to confirm the results. Platelet volume can reflect platelet function and activation degree (38). MPV is inversely correlated with platelet count (39). A previous study (40) reported that large platelets increased in the course of an inflammatory condition. Simultaneously, these cells were rapidly worn by the inflammation (41). This seems to explain the drop in platelet count and the rise in MPV in patients with ongoing acute schizophrenia in our study. NLR, MLR, and PLR are convenient markers of the systemic inflammatory response that seem to be less affected by confounding conditions, and provide more stable information than other single leukocyte parameters (42). Previous reports (18, 23) have also shown that NLR, PLR, and MLR values were higher in patients with schizophrenia than in healthy controls. Consistent with previous studies, our study found that levels of NLR and MLR in acute schizophrenia patients were increased compared with HCs. Unexpectedly, we found that PLR is lower in patients with schizophrenia than healthy controls. Yu et al. (34) conducted a study in 106 first-episode schizophrenia patients and found that PLR is higher than in healthy controls. Ozdin and Boke (32) studied 105 relapse hospitalized schizophrenia patients and found that PLR is higher for the sample than HCs. All these opposite results were obtained in small sample sizes. The sample size of our study is larger, which could provide more reliable supports for the conclusion that PLR is lower in patients with acute psychotic episodes of schizophrenia than in HCs. However, the results of our study could be influenced by antipsychotics, so further study is needed to purify the samples to further verify the results. RDW as a reflecting variability marker of erythrocyte volume is a routinely available component of CBC. Several studies have suggested inflammation as a possible cause of elevated RDW (18). However, the underlying biological mechanisms is still unclear (43). Balcioglu and Kirlioglu's research (18) reported higher levels of RDW in schizophrenia than in HCs, which is consistent with our results. Above all, we speculate that the inflammation occurring in schizophrenia could lead to consumption of bilirubin during scavenging of excessive free radicals, causing a consequent reduction in their serum levels. The ongoing apoptotic effect of UCB on lymphocyte and platelet, which has been shown in other studies, may be the mechanism through which UCB affects inflammatory in acute stage schizophrenia. All the relationships are weak in our study. We speculate that the mechanism of UCB protecting individuals from schizophrenia is complex including inflammatory processes, immune processes may also be involved (44), and we should pay more attention to UCB of schizophrenia, which could provide some clues to the mechanism of schizophrenia and facilitate finding new ways of protecting schizophrenia.

In an attempt to identify patients with schizophrenia in the acute stage, several predictors of biomarkers have been investigated. In Ozdin and Boke's study (32), MLR and PLR were found to be important markers in schizophrenia. Balcioglu and Kirlioglu (18) uses logistic regression analysis to demonstrate a significant predictive diagnosis value of MPV in schizophrenia. In addition to inflammation, oxidative stress plays a key role in the progression of schizophrenia, while bilirubin is a strong endogenous antioxidant (45). A systematic review (25) showed that UCB is in part implicated in the pathophysiological process of schizophrenia, and elevated bilirubin may be linked with lower risk of schizophrenia in the acute stage. One recent study (46) showed potential of UCB as a biological marker for schizophrenia. Nevertheless, to our knowledge, the present study is the first to indicate that the combined effect of peripheral biomarkers of inflammation derived from CBC and UCB could be better diagnosis parameters compared with single predictor independently in patients with acute schizophrenia. According to the logistic regression analysis, UCB and PLR play a role in the protection against the incidence of schizophrenia of acute stage, and MLR and MPV are risk factors for acute schizophrenia. From our results, UCB has potential diagnostic value, especially when combined with inflammatory indicators. Further study may focus on this point to help with the lack of objective diagnostic criteria for schizophrenia. Further studies should pay attention to the change of UCB and PLR, MLR, and MPV, which may be prognostic biomarkers.

## Limitations of the Study

This study aims to leverage the advantage of a large sample of acute episode schizophrenia subjects on complete blood cell count parameters and unconjugated bilirubin. However, as a retrospective study, there is a lack of clinical information, such as illness duration and PANSS scores, during the hospitalization period of the patients and potential confounding factors such as antipsychotic treatment, including dosage, time on treatment, smoking, and BMI of subjects. As a cross-sectional study, our study lacks conclusions regarding causal relationships between UCB and inflammation as well as relationships between UCB, inflammation, and schizophrenia. Additional markers of platelet activation are also required (i.e., P-selectin) to further verify the significance of these findings.

## Conclusion

In this study, we found that there is an increase in blood total WBC counts, neutrophils, monocytes, NLR, MLR, and RDW, and a reduction in lymphocyte counts, platelet counts, PLR, and UCB in schizophrenia of acute stage. We have also observed a negative correlation between UCB and lymphocyte, platelet, MPV, and RDW, and a positive correlation between UCB and NLR. All the relationships are weak; hence, we speculate that UCB has a protection effect on acute stage of schizophrenia, which is weak and indirect by affecting the proinflammatory processes. Other potential mechanisms of the protection effect of UCB in schizophrenia need to be explored further. Our findings suggest that a combination of markers including MLR, MPV, PLR, and UBC could be used to predict biomarkers of acute schizophrenia.

## Data Availability Statement

The original contributions presented in the study are included in the article/supplementary material, further inquiries can be directed to the corresponding authors.

## Author Contributions

HX: conceptualization, data curation, formal analysis, investigation, and writing—original draft. YW: conceptualization, methodology, validation, investigation, writing—review, and editing. LZ and TL: data curation, methodology, supervision, and validation. HL and JM: conceptualization, project administration, supervision, writing—review, and editing. JC: funding acquisition, conceptualization, project administration, supervision, writing—review, and editing. All authors contributed to the article and approved the submitted version.

## Funding

This work was funded by Beijing Hospitals Authority Clinical Medicine Development, Scientific Foundation of Beijing Huilongguan Hospital, No LY202204, and Beijing Municipal Administration of Hospitals incubating Program (PX 2022079).

## Conflict of Interest

The authors declare that the research was conducted in the absence of any commercial or financial relationships that could be construed as a potential conflict of interest.

## Publisher's Note

All claims expressed in this article are solely those of the authors and do not necessarily represent those of their affiliated organizations, or those of the publisher, the editors and the reviewers. Any product that may be evaluated in this article, or claim that may be made by its manufacturer, is not guaranteed or endorsed by the publisher.
